# 424. High Prevalence of Neuropsychiatric Symptoms and Pre-COVID-19 Mental Health Disorders in Patients with Post-Acute COVID

**DOI:** 10.1093/ofid/ofad500.494

**Published:** 2023-11-27

**Authors:** Nassim Mokraoui, Sara Brenner, Amanda Westlake, Vida Rastegar, Daniel Skiest

**Affiliations:** UMass Chan Baystate Medical Center, Champaign, Illinois; UMass Chan Baystate Medical Center, Champaign, Illinois; UMass Chan Baystate Medical Center, Champaign, Illinois; UMass Chan Baystate Medical Center, Champaign, Illinois; UMass Chan Baystate Medical Center, Champaign, Illinois

## Abstract

**Background:**

Individuals with post-acute COVID syndrome (PAC), also known as long-COVID, have new or continuing symptoms lasting more than 1 month from COVID-19 infection. Patients may present with neuropsychiatric (NP) and non-NP symptoms. Whether prior mental health disorders correlate with NP symptoms is unknown. The study aims were to describe the demographics, clinical factors, comorbid conditions, and presenting symptoms of PAC.

**Methods:**

We included patients evaluated at Baystate Health PAC clinic from January 2021-April 2022, with prior COVID-19 infection and new or persistent symptoms of at least 6 weeks. We defined the primary presenting symptoms as NP (cognitive dysfunction (brain fog), anxiety, or depression) or non-NP (dyspnea, fatigue, dizziness, palpitations). The clinician’s diagnostic impression was recorded and characterized as NP or non-NP.

Demographics

**Figure:**
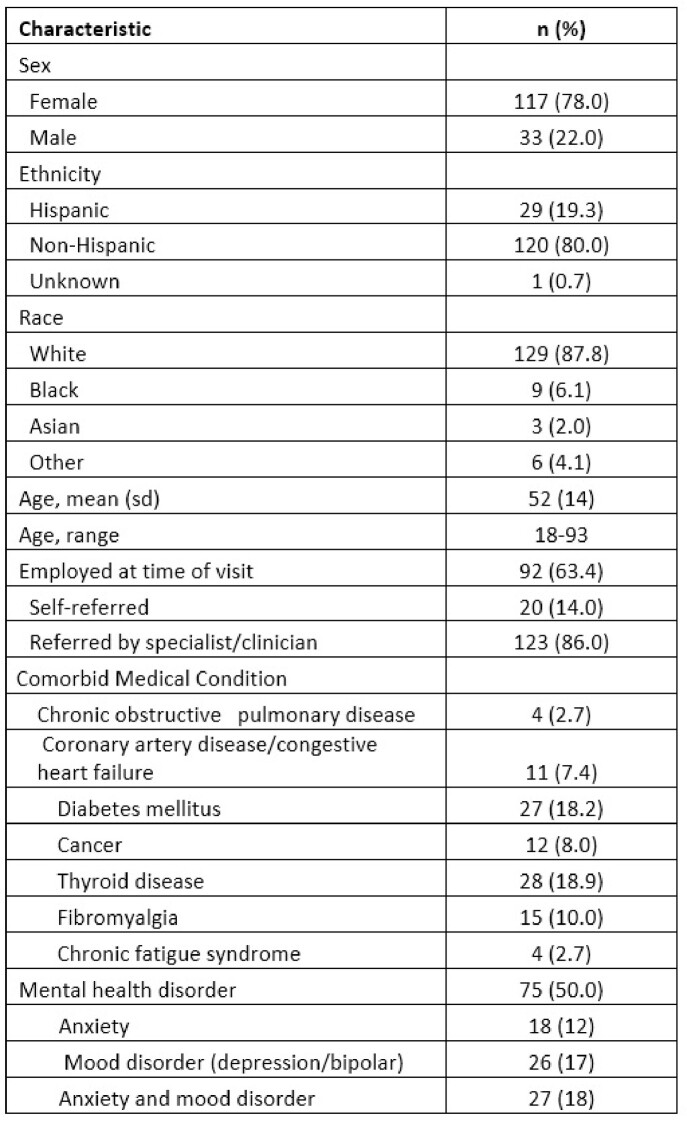

**Results:**

We randomly selected 150 of 250 clinic patients for chart review. Most were women, 117 (78%); mean age was 52. Most 106 (71%) had mild acute COVID illness; 36 (24%) were hospitalized. 49% (n=73) had a pre-existing mental health disorder: mood disorder (depression/bipolar) 26 (17%), anxiety 18 (12%), both anxiety and mood disorder 27 (18%). The most common presenting symptoms in order of frequency were fatigue, dyspnea, brain fog, anxiety, depression, and palpitations. 106 (71%) had at least 1 NP presenting symptom or combination of NP symptoms; cognitive dysfunction with anxiety in 23 (15%), and cognitive dysfunction with anxiety and mood disorder in 13 (9%). The clinician's final diagnostic impression was fatigue (47%), cognitive dysfunction (36%), anxiety (29%) depression (33%) and PTSD (3%). A NP diagnosis was made in 81 (54%).

Primary Symptoms

**Figure:**
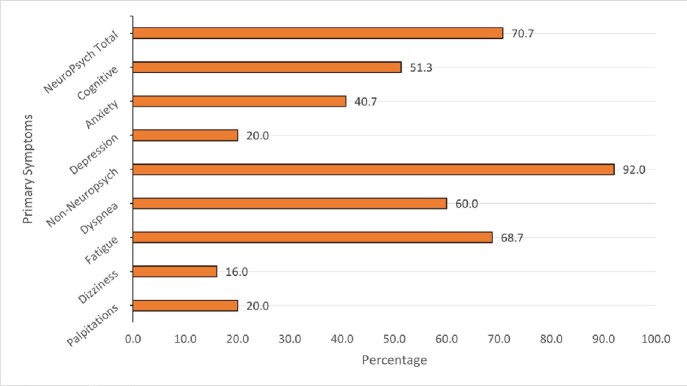

Primary Diagnosis

**Figure:**
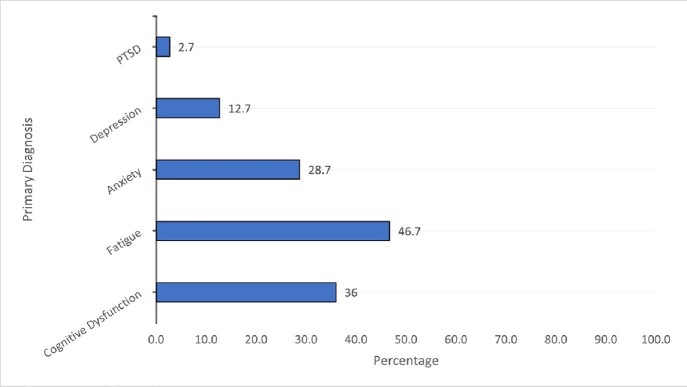

**Conclusion:**

The majority of PAC clinic patients were female and had mild COVID illness. We noted a high prevalence of pre-existing mental health disorders. More than two-thirds presented with one or more NP symptom. Further studies should explore the association of pre-COVID mental health disorders with PAC.

**Disclosures:**

**All Authors**: No reported disclosures

